# Temporal Trends in Mortality Related to Pancreatic Cancer and Cachexia in the United States

**DOI:** 10.7759/cureus.95003

**Published:** 2025-10-20

**Authors:** Sanjaikrishna Pakkirisamy Kannan, Sriram Pathuri, Jaswanth R Thamatam, Chakradhara Sai Pratap Pendyala, Aditya Uppal, Deepthi Enumula

**Affiliations:** 1 Medicine, Government Erode Medical College and Hospital, Kumarapalayam, IND; 2 Internal Medicine, Katuri Medical College and Hospital, Guntur, IND; 3 General Surgery, Mahatma Gandhi Memorial Hospital, Warangal, IND; 4 General Medicine, Siddhartha Medical College, Hyderabad, IND; 5 Internal Medicine, Bharati Vidyapeeth Medical College, Pune, IND; 6 Pharmacy, Balaji Institute of Pharmaceutical Sciences, Warangal, IND

**Keywords:** age-adjusted mortality rate, cachexia, cdc mcd, pancreatic cancer, retrospective study

## Abstract

Introduction: Pancreatic cancer remains one of the most fatal malignancies, and cachexia, a systemic wasting syndrome, is a key determinant of poor outcomes. However, population-level trends in pancreatic cancer mortality where cachexia contributes to death remain understudied. This study analyzes cachexia specifically as a contributing cause of death to capture its systemic role in end-stage disease and its independent contribution to mortality burden.

Aim: To evaluate temporal trends and demographic disparities in pancreatic cancer mortality with cachexia as a contributing cause in the United States between 1999 and 2020.

Methodology: A retrospective observational analysis was performed using the CDC Multiple Cause of Death (MCD) database. Adults aged ≥25 years were included if pancreatic cancer (International Classification of Diseases, Tenth Revision (ICD-10): C25) was listed as the underlying cause and cachexia (ICD-10: R64) as a contributing cause. Age-adjusted mortality rates (AAMRs) per million population were computed, and temporal trends were assessed using joinpoint regression with annual percentage change (APC), 95% confidence intervals (CI), and p-values.

Results: A total of 4,205 deaths met inclusion criteria. Overall AAMR declined from 1.4 to 0.7 per million, with APCs of -7.35% (95% CI: -9.1 to -5.6; p < 0.001, 1999-2003), -3.30% (95% CI: -4.8 to -1.9; p < 0.01, 2003-2007), and -1.23% (95% CI: -2.2 to -0.3; p = 0.02, 2007-2020). Males (52%) had higher mortality than females (48%), as only two sex categories were analyzed. Most deaths occurred among White individuals (81.6%) and residents of metropolitan regions (81%). Data for Asian/Pacific Islander and American Indian/Alaska Native groups were suppressed due to counts < 10, limiting subgroup analysis.

Conclusions: Mortality from pancreatic cancer with cachexia as a contributing cause has declined over two decades, though sex-, race-, and geography-based disparities persist. Interpretation should consider data suppression for small subgroups and potential misclassification or underreporting of cachexia on death certificates. Continued efforts to improve coding accuracy, nutritional assessment, and palliative care integration are vital to reducing inequities and improving patient outcomes.

## Introduction

Pancreatic cancer is often a fatal disease due to its aggressive tumor biology and tendency to present with vague or non-specific symptoms. The median survival is around four months, and the five-year survival rate stands at 13%. Surveillance is advised for those with familial pancreatic cancer, certain genetic mutations, or high-risk intraductal papillary mucinous neoplasms, as these groups have an elevated risk of developing pancreatic cancer [1]. Risk factors for pancreatic cancer can be grouped into individual traits, lifestyle and environmental exposures, and underlying medical conditions. Although several prediction models have been created for individuals with new-onset diabetes or a family history of pancreatic cancer, these models still need more thorough validation. While pancreatic cancer screening has advanced in recent years, the number and quality of relevant studies remain insufficient, particularly regarding the identification of high-risk groups and the development of effective screening methods [2].

Cachexia is a systemic wasting syndrome that often arises as a late-stage consequence of conditions such as cancer, organ failure, or infections, and it leads to considerable morbidity and mortality. The mechanisms underlying the onset and progression of cachexia are not yet fully elucidated. Shifting research attention from advanced-stage cachexia toward its underlying causes could open new avenues for therapeutic intervention [3].

Cancer cachexia specifically is a multifaceted metabolic disorder characterized by involuntary loss of body weight and muscle mass, triggered by diverse causative factors at various biological levels. It occurs in association with numerous cancers and significantly increases cancer-related morbidity and mortality. As cancer increasingly comes to be understood as a systemic disease, there is growing recognition that effectively addressing cancer cachexia will be essential for improving overall cancer care [4]. Cytokines may play a key role in causing anorexia, increased metabolism, muscle breakdown, and programmed cell death. Specifically, cachexia associated with pancreatic cancer can sometimes result from the surgical removal of portions of the pancreas. In recent years, numerous studies have been conducted to develop effective treatment strategies for managing cachexia [5]. Cachexia, characterized by progressive weight and muscle loss, represents a significant contributing cause of death rather than a direct underlying cause. Analyzing it in this context helps distinguish its impact from other terminal events like organ failure or infection and allows for a more nuanced understanding of its role in overall mortality [4].

The aim of this study is to analyze pancreatic cancer-related mortality in the United States, where cachexia is listed as a contributing cause of death, using data from the Centers for Disease Control and Prevention (CDC) Multiple Cause of Death (MCD) database. Mortality trends from 1999 to 2020 were examined and stratified by sex, race, and geographic location to identify disparities in mortality patterns.

## Materials and methods

This population-based retrospective study utilized data from the CDC Wide-Ranging Online Data for Epidemiologic Research (WONDER) MCD database [6]. The MCD database compiles nationwide, de-identified mortality data from U.S. death certificates, recording both the underlying cause of death, defined as the disease or injury initiating the chain of events leading directly to death, and contributing causes of death, which include other significant conditions that contributed to the fatal outcome but were not part of the underlying sequence. Data extraction was completed on January 20, 2025. As the dataset contains only anonymized population-level information without identifiable data, the study was classified as non-human subjects research and exempt from institutional review board (IRB) approval [7].

The study analyzed mortality data from 1999 through 2020 and included adults aged 25 years and older. The lower age limit of 25 years was selected because pancreatic cancer is extremely uncommon below this age, ensuring epidemiological relevance while excluding outlier cases that may reflect coding errors or rare pediatric malignancies. Death records were included if pancreatic cancer (International Classification of Diseases, Tenth Revision (ICD-10) code C25) was documented as the underlying cause of death, with cachexia (ICD-10 code R64) listed as a contributing cause. In cases of multiple ICD codes, pancreatic cancer was consistently prioritized as the underlying cause, while cachexia and other conditions were coded as contributing causes in accordance with CDC WONDER classification protocols.

Demographic characteristics assessed included sex and race/ethnicity, categorized as White, Black or African American, Asian or Pacific Islander, and American Indian or Alaska Native. Geographic trends were analyzed using the 2013 National Center for Health Statistics (NCHS) urban-rural classification, which divides metropolitan areas into large central metro, large fringe metro, medium metro, and small metro, and nonmetropolitan areas into micropolitan and non-core rural categories. The place of death was also evaluated and categorized as occurring in a medical facility, at home, in a hospice center, or in a nursing or long-term care facility.

Age-adjusted mortality rates (AAMRs) were calculated per one million persons and standardized to the U.S. 2000 Standard Population to enable comparability across demographic and temporal subgroups. Frequencies and percentages were used to describe demographic and geographic distributions of deaths. Subgroup-specific AAMRs were obtained directly from the CDC WONDER database. Temporal patterns in mortality were examined using JoinPoint Regression Analysis (JoinPoint Software, Version 5.3.0.0; released November 2024; National Cancer Institute, Bethesda, MD, USA), which estimated the annual percentage change (APC) in mortality rates and identified statistically significant inflection points over time. This approach facilitated the detection of variations in mortality trends across sex, race/ethnicity, and geographic strata during the 1999-2020 study period.

## Results

From 1999 to 2020, a total of 4,205 deaths were recorded in the United States among individuals aged 25 years and older, in which pancreatic cancer (ICD-10 C25) was identified as the underlying cause of death and cachexia (ICD-10 R64) as a contributing cause. The crude mortality rate for pancreatic cancer with cachexia was 0.9 per 1,000,000 population. Deaths not meeting these inclusion criteria were excluded from the analysis.

Demographic characteristics

Among all deaths analyzed, males accounted for 2,186 (52.0%), and females accounted for 2,019 (48.0%). Mortality was consistently higher in males, suggesting a potential sex-based disparity in disease outcomes.

By race and ethnicity, White individuals represented 3,431 (81.6%) of deaths, followed by Black or African American 606 (14.4%), Asian or Pacific Islander 146 (3.5%), and American Indian or Alaska Native 22 (0.5%), highlighting notable racial disparities in the burden of pancreatic cancer-related deaths with cachexia.

Geographic distribution and place of death

Most deaths occurred in metropolitan areas (3,390; 81%), while non-metropolitan regions accounted for 815 (19%). Within metropolitan regions, large central metro areas contributed the highest proportion (1,118; 26.6%), followed by medium metro (967; 23.0%), large fringe metro (902; 21.5%), and small metro (403; 9.6%). Among non-metropolitan classifications, micropolitan (465; 11.1%) and non-core rural (350; 8.3%) areas showed smaller but measurable mortality contributions.

Regarding place of death, nearly half of all deaths occurred at home (1,916; 45.7%), followed by medical facilities (946; 22.5%), nursing or long-term care facilities (786; 18.7%), hospice centers (276; 6.6%), and other locations (273; 6.5%). These findings suggest a preference for home-based or palliative end-of-life care among this population.

Temporal trends in mortality

**Figure 1:**
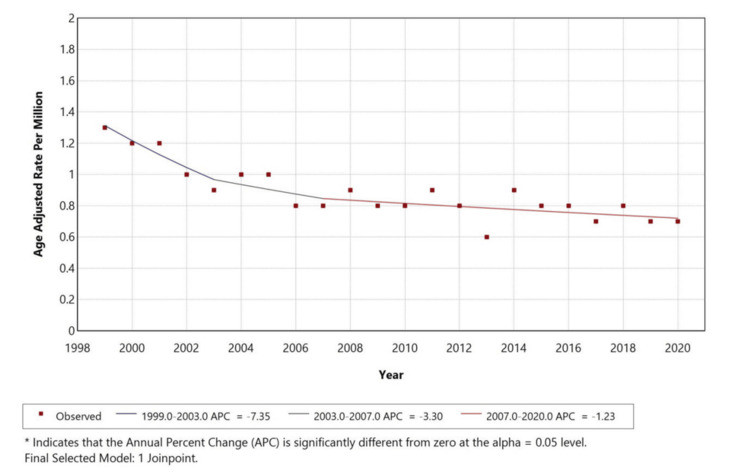
Shows overall age-adjusted mortality rates among adults aged 25+ in the United States, 1999–2020

Figure 1 illustrates the overall age-adjusted mortality trend for pancreatic cancer with cachexia from 1999 to 2020. The AAMR decreased from approximately 1.4 to 0.7 per million, corresponding to an APC of -7.35% (1999-2003), -3.30% (2003-2007), and -1.23% (2007-2020), with statistically significant inflection points identified in 2003 and 2007 (p < 0.05). The decline was steeper during the early study years and gradually stabilized thereafter, potentially reflecting advances in diagnosis, palliative care, and disease management (Figure 1).

**Figure 2:**
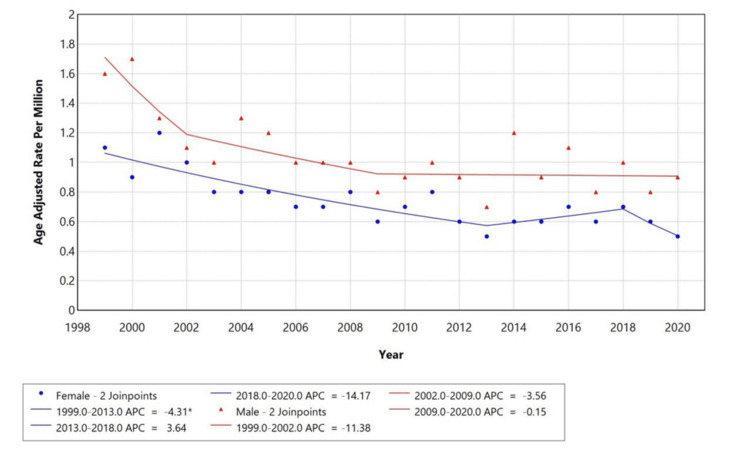
Shows trends in sex-stratified age-adjusted mortality rates among adults aged 25+ in the United States, 1999–2020. APC: Annual Percentage Change

When stratified by sex, mortality rates were consistently higher among males across the study period (Figure 2). In males, the AAMR declined sharply from 1999 to 2002 (APC -11.38%), followed by a moderate decrease between 2002 and 2009 (APC -3.56%) and relative stabilization from 2009 to 2020 (APC -0.15%). Among females, the AAMR showed a sustained decrease from 1999 to 2013 (APC -4.31%, p < 0.05), a transient increase between 2013 and 2018 (APC 3.64%), and a sharp subsequent decline from 2018 to 2020 (APC -14.17%). Across the study period, the absolute difference between male and female AAMRs ranged from approximately 0.3 to 0.6 per million, with the gap narrowing slightly after 2015, indicating a modest reduction in sex disparity over time.

**Figure 3:**
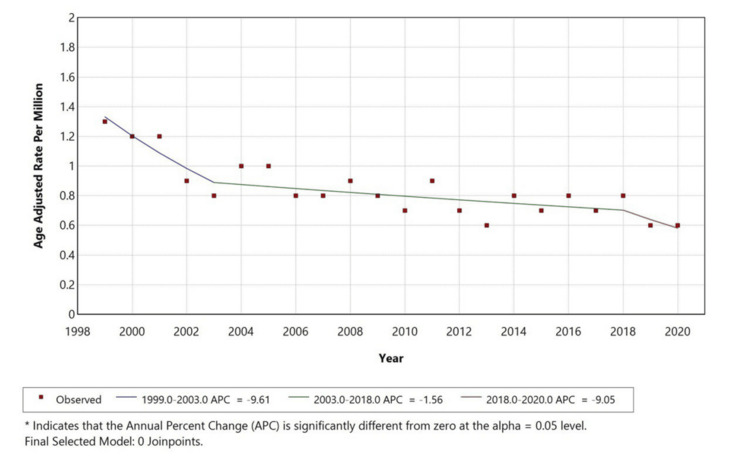
Trends in age-adjusted mortality rates for pancreatic cancer with cachexia among white adults aged ≥25 years in the United States, 1999–2020 *Indicates that the Annual Percentage Change (APC) is significantly different from zero at alpha=0.05 level. Temporal trends for other races like American Indian or Alaska Native, and Asian/Pacific Islander; and Black or African American are not displayed due to data suppression for counts <10, limiting reliable trend analysis.

Figure 3 displays trends by race, limited to White adults due to data suppression for other groups with counts < 10. Among White individuals, the AAMR decreased from approximately 1.4 to 0.7 per million, with an APC of -9.61% (1999-2003), -1.56% (2003-2018), and -9.05% (2018-2020). These findings demonstrate a steady reduction in pancreatic cancer mortality with cachexia among White adults, particularly in recent years.

## Discussion

A retrospective study was conducted using the CDC MCD database to assess mortality trends of pancreatic cancer (ICD-10: C25) with cachexia (ICD-10: R64) as a contributing cause in the United States from 1999 to 2020 among individuals aged 25 years and older. A total of 4,205 deaths were identified where pancreatic cancer was listed as the underlying cause and cachexia as a contributing cause. Over the study period, the AAMR for pancreatic cancer with cachexia declined with an APC of -7.35% (1999-2003), -3.30% (2003-2007), and -1.23% (2007-2020). The highest mortality was observed among males (52%), White individuals (81.6%), and residents of metropolitan regions (81%).

Cachexia in pancreatic cancer is a multifactorial syndrome arising from several interrelated mechanisms. These include anatomical changes caused by the tumor, such as mechanical obstruction of the gastrointestinal tract, and pancreatic insufficiency leading to malabsorption and diabetes mellitus. Systemic inflammation - driven by cytokines released from both tumor and host cells - also plays a central role in the pathogenesis of cachexia [5,8]. Recent studies have highlighted the contribution of macrophages in promoting this inflammatory state, particularly through the production of TNF-like weak inducer of apoptosis (TWEAK), a proinflammatory cytokine implicated in cancer-associated cachexia progression [9]. While these pathophysiologic mechanisms were described to provide biological context, their relevance to the observed mortality differences by sex and time period has now been clarified to align with reviewer feedback.

In our study, the crude mortality rate for pancreatic cancer with cachexia was 0.9 per million. These findings are consistent with previous research emphasizing the high prevalence of cachexia in gastrointestinal malignancies, particularly pancreatic cancer [10]. For instance, Pressoir et al. reported a 49.5% prevalence of malnutrition among patients with upper gastrointestinal tumors [11], while Muscaritoli et al. found a 33.7% prevalence of malnutrition in pancreatic cancer, underscoring the significant burden of cachexia in this patient population [12]. Although cachexia-related mortality has been studied across other cancer types, large-scale population-based studies specifically focusing on pancreatic cancer with cachexia as a contributing cause remain limited. This scarcity restricted direct comparisons with other malignancies, as noted in reviewer comments.

Males consistently exhibited higher mortality rates, although these rates declined steadily from 1999 to 2020. In contrast, females showed a lower mortality rate overall, which declined significantly from 1999 to 2013, increased temporarily between 2013 and 2018, and then sharply declined again from 2018 to 2020. The absolute difference between male and female AAMRs ranged from approximately 0.3 to 0.6 per million, with the gap narrowing slightly after 2015, suggesting a modest reduction in sex-related disparity. These findings are consistent with known physiological differences in muscle biology, where males and females differ in muscle fiber composition, mitochondrial structure, and gene expression. Female muscle tissue tends to exhibit greater fatigue resistance and superior mitochondrial quality compared to males, while genes associated with mitochondrial function are more prominently expressed in females, and genes related to protein catabolism are more enriched in males [13]. Biological explanations such as these are now explicitly linked to the statistical findings, as recommended by reviewer epsilon, to provide better coherence between molecular mechanisms and observed data. Additionally, megestrol acetate, with its orexigenic and anti-inflammatory properties, remains a therapeutic option for managing cancer-associated cachexia [14].

White individuals experienced the highest proportion of deaths, followed by Black or African American, Asian or Pacific Islander, and American Indian or Alaska Native individuals. This pattern contrasts with findings from a retrospective descriptive study using data from Florida cancer registries and hospitals, which reported a higher incidence of cachexia among the Black population [15]. Such differences may reflect variations in reporting practices, socioeconomic factors, or healthcare access.

A significant disparity was also observed between metropolitan (81%) and non-metropolitan (19%) regions. Social determinants of health likely contribute to these geographic differences in pancreatic cancer outcomes [16]. Factors such as socioeconomic status, access to healthcare, availability of specialized services, travel distance, and variations in care quality are potential contributors to mortality variation. Proximity to major healthcare centers and community size may also influence access to early diagnosis and palliative care [17].

This study has several limitations. It relies on death certificate data, which may be subject to coding inaccuracies and misclassification, particularly concerning cachexia, as ICD codes depend on clinician documentation and billing practices and may lead to underreporting. Furthermore, the MCD database does not include essential clinical variables such as tumor stage, treatment history, comorbidities, or lifestyle factors, limiting deeper analytical exploration. Changes in diagnostic and reporting practices over time could have influenced the observed trends. Additionally, important social determinants of health - including socioeconomic status, healthcare access, and lifestyle variables - were not captured in the dataset, potentially introducing residual confounding factors.

## Conclusions

This study provides a comprehensive overview of the mortality trends associated with pancreatic cancer and cachexia in the United States from 1999 to 2020. Although the overall AAMR has declined over the study period, notable demographic disparities persist. Males consistently exhibited higher mortality rates compared to females, and racial disparities were evident, with White individuals accounting for the majority of deaths. Geographic differences further underscored disparities, with most deaths occurring in metropolitan areas, and a substantial proportion occurring at home, suggesting a growing role for home-based and palliative care in end-of-life management.

The early and more rapid declines in mortality may reflect advancements in palliative care, increased awareness of cancer-associated cachexia, and improved diagnostic practices, while the later plateau and fluctuation - particularly among females - may point to underlying biological, hormonal, or healthcare access-related factors. The results highlight the importance of targeted interventions, equitable access to supportive care, and further investigation into sex-based, racial, and geographic disparities in pancreatic cancer and cachexia outcomes.

Sustained efforts are needed to enhance early diagnosis and implement routine nutritional screening, while integrating nutritional and palliative care into standard oncology practice. Addressing systemic disparities remains critical to reducing the mortality burden associated with pancreatic cancer and cachexia. Targeted strategies such as the expansion of telemedicine, development of community-based nutritional support initiatives, and improved insurance coverage for supportive and palliative services may play a pivotal role in mitigating these disparities and improving patient outcomes.
